# Applications of DNA barcoding to fish landings: authentication and diversity assessment

**DOI:** 10.3897/zookeys.365.6409

**Published:** 2013-12-30

**Authors:** Alba Ardura, Serge Planes, Eva Garcia-Vazquez

**Affiliations:** 1 University of Oviedo, Department of Functional Biology. C/ Julian Claveria s/n. 33006-Oviedo, Spain; 2 USR 3278 CNRS – EPHE. Centre de Recherche Insulaire et Observatoire de l’Environnement (CRIOBE) BP 1013 - 98 729, Papetoai, Moorea, Polynésie française; 3 Centre de Biologie et d’Ecologie Tropicale et Méditerranéenne, Université de Perpignan, 52 Av. Paul Alduy - 66860 Perpignan cedex, France

**Keywords:** Species identification, freshwater fisheries, marine fisheries, genetic diversity, mitochondrial DNA markers

## Abstract

DNA barcoding methodologies are being increasingly applied not only for scientific purposes but also for diverse real-life uses. Fisheries assessment is a potential niche for DNA barcoding, which serves for species authentication and may also be used for estimating within-population genetic diversity of exploited fish. Analysis of single-sequence barcodes has been proposed as a shortcut for measuring diversity in addition to the original purpose of species identification. Here we explore the relative utility of different mitochondrial sequences (12S rDNA, COI, cyt *b*, and D-Loop) for application as barcodes in fisheries sciences, using as case studies two marine and two freshwater catches of contrasting diversity levels. Ambiguous catch identification from COI and cyt *b* was observed. In some cases this could be attributed to duplicated names in databases, but in others it could be due to mitochondrial introgression between closely related species that may obscure species assignation from mtDNA. This last problem could be solved using a combination of mitochondrial and nuclear genes. We suggest to simultaneously analyze one conserved and one more polymorphic gene to identify species and assess diversity in fish catches.

## Introduction

DNA barcoding is increasingly important in natural sciences. For ecologists it is a tool with many utilities (e.g. Valentini et al. 2009), most of which are related with biodiversity inventories. Fisheries are a field of enormous potential interest for barcoding applications. The use of genetics is increasingly required in fisheries for species authentication in fish landings (Rasmussen and Morrisey 2008, Ardura et al. 2010a). Fisheries are unsustainable if catch records are based on erroneous or inaccurate species identifications (Watson and Pauly 2001, Marko et al. 2004, Crego et al. 2012). Moreover, guaranteeing species authenticity along the commercial chain would improve consumer’s security and prevent fraud, which has been proven to occur worldwide (e.g. DeSalle and Birstein 1996, Marko et al. 2004, Jacquet and Pauly 2008, Wong and Hanner 2008, Ardura et al. 2010b, Ardura et al. 2010c, Barbuto et al. 2010, Filonzi et al. 2010, Miller and Mariani 2010, Garcia-Vazquez et al. 2011). On the other hand, declines in population genetic variation diminish the ability of a population to adapt to environmental changes and decrease its chance of long-term survival (Frankham 1995, Hedrick 2001, Wang et al. 2002); thus periodical monitoring of population variation of exploited stocks is highly recommended in fisheries management.

Despite the potential importance of genetics in fisheries, the application of DNA analyses in real cases is not so easy. The economic aspect is crucial: increasing costs are making fisheries not only ecologically, but also economically unsustainable (e.g. Willmann and Kelleher 2010). The practical use of genome-wide studies in everyday management does not seem to be realistic in a near future because massive DNA analysis of catches would increase even more the costs of fish products. If the genetic tool (marker) employed for species authentication exhibits enough variation for reliable quantification of population diversity, a single analysis could solve two problems at the same time. Another practical problem for applying genetics to fisheries is the time required for DNA analysis. Catches can not be immobilized for a long time without increasing storage costs for guaranteeing the cold chain. The accelerated development of high throughput sequencing methodologies (e.g. Steemers and Gunderson 2005, Sundquist et al. 2007) can help in this issue because now it is possible to analyze thousands of samples very fast. Genomics at population level is being carried out for a few targeted marine species (Nielsen et al. 2009); the moment of applying large scale routine genetic analysis in fisheries science, including all species, seems thus to be approaching.

The potential taxonomic diversity of fish catches is enormous, since in biodiversity hotspots unknown species are landed (Worm and Branch 2012). This makes it difficult to analyze introns and SNP of the nuclear genome, whose development requires a good knowledge of each species’ genome for developing primers in flanking regions. However, using universal primers is much easier. Demographic changes in fish populations can be associated with the observed amount of variation in mitochondrial DNA (e.g. Fauvelot et al. 2003, Nevado et al. 2013), and genetic erosion due to population depletion could be theoretically detected from variable mitochondrial regions. The international barcoding initiative (Hebert et al. 2003, Janzen et al. 2005) has converged with next-generation sequencing, and ecosystem biodiversity can be better estimated through DNA information now (Hajibabei 2012). The main DNA barcode has been chosen by some authors as an initial tool for calibrating fish species diversity due to the large number of cytochrome *c* oxidase I gene (COI) sequences included in the *Barcode of Life Data Systems* (BOLD) database (April et al. 2011, Ardura et al. 2011). However, it may not be sufficient to rigorously address intraspecific variation at population level (Moritz and Cicero 2004, Rubinoff 2006). The informative value of other DNA regions with different degrees of polymorphism should therefore be evaluated. The highly conserved mitochondrial 12S rDNA has been applied for analyzing diversity in high categorical levels such as phyla (Gerber et al. 2001). In decreasing order of conservation, the protein-coding cytochrome *b* (cyt *b*) has been extensively used for diversity analysis at genera and species level (Min et al. 2004, Zhang and Jiang 2006). Finally, the D-Loop or mitochondrial control region exhibits more variation than protein-coding sequences due to reduced functional constraints and relaxed selection pressure (Onuma et al. 2006, Wu et al. 2006). Therefore, D-Loop variation would roughly inform about intraspecific diversity, whereas more conserved sequences would better reflect biodiversity (number and genetic proximity of species in a catch).

The objective of this study was to assess the utility of well-known public databases for identifying catches from very different fisheries, comparing genes and species for determining if there is sufficient information available for routine genetic analysis of fish catches that informs about species composition. The main areas where generating new data are necessary, if any, will be identified from the shortcomings detected in this small-scale exercise. We have employed standard primer sets for PCR amplification of four mtDNA gene fragments, then estimated standard parameters of genetic diversity and evaluated their utility for identifying landings using GenBank and BOLD. We have also estimated intrapopulation diversity in order to assess possible applications of these markers for monitoring demographic changes. Our case studies were two marine and two freshwater catches of contrasting diversity for the standard COI DNA barcode (Ardura et al. 2011).

## Materials and methods

### Case studies

Mediterranean Sea. It is a marine biodiversity hotspot with 713 fish species inventoried (FishBase; www.fishbase.org). Samples were obtained from fish markets in the Languedoc-Roussillon region (Gulf of Lion, France), in the north-western Mediterranean coast.

Cantabric Sea. Less diverse than the Mediterranean Sea, it contains 148 fish species inventoried. Catch from commercial fisheries was sampled from fish markets in Asturias (North of Spain).

Amazon River. It is the main freshwater biodiversity hotspot of the world (1218 inventoried fish species). We have sampled catches obtained in different fish markets of Manaus (Brazil). This is the area where the two main Amazonian drainages (the rivers Negro and Solimões) join.

Narcea River (North of Spain). As other North Iberian rivers, it exhibits reduced biodiversity with only 17 fish species inventoried. Fisheries are strongly targeted and focused on sport angling of salmonids. Samples were obtained *in situ* from fishermen in the lower reach of the river.

The two most exploited species (those that yield more tonnes in official catch statistics) from each site were chosen for this study. They were: mackerel *Scomber scombrus* (Goode, 1884) and anchovy *Engraulis encrasicolus* (Linnaeus, 1758) from the Mediterranean Sea; mackerel and albacore tuna *Thunnus alalunga* (Bonnaterre, 1778) from the Cantabric Sea; Curimatá *Prochilodus nigricans* (Spix & Agassiz, 1829) and jaraquí *Semaprochilodus insignis* (Jardine & Schomburgk, 1841) from the Amazon River; Atlantic salmon *Salmo salar* (Linnaeus, 1758) and brown trout *Salmo trutta* (Linnaeus, 1758) from the Narcea River. These species do not exhibit population sub-division in the fishing areas considered. The West Mediterranean and the Eastern Atlantic Ocean populations of mackerel seem to form a panmictic unit (Zardoya et al. 2004). The highly migratory albacore tuna exhibits only inter-oceanic population differentiation or between the Atlantic and the Mediterranean, not within the same ocean (Chow and Ushiama 1995, Viñas et al. 2004). For anchovy, the whole north-western Mediterranean likely harbors a single population (Tudela et al. 1999). Curimatá and jaraquí, the main catch in the Brazilian Amazon state, have a shallow genetic structuring in the Amazon basin and can be considered homogeneous populations around Manaus (Ardura et al. 2013). Finally, Atlantic salmon and brown trout populations are not subdivided within rivers in North Spain unless there is strong habitat fragmentation (e.g. Horreo et al. 2011a, b), yet this is not the case for the lower accessible zone of River Narcea.

Ten samples were analyzed per species.

### mtDNA analysis

DNA extraction was automatized with QIAxtractor robot following the manufacturer’s protocol (QIAGEN DX Universal DNA Extraction Tissue Sample CorProtocol), which yields high quality DNA suitable for a wide variety of downstream applications. The procedure is divided into two sections: digestion and extraction. The digestion process favors tissue dissociation and liquid suspension, and is ready for extraction.

Briefly, a 96 well round well lysis block (Sample Block) is loaded with 420 µl DX Tissue Digest (containing 1% v/v DX Digest Enzyme) manually or using the Tissue Digest Preload run file. Once the DX Tissue Digest is loaded with the sample, the sample block is sealed and incubated at 55 °C with agitation for at least 3 h. 220 µl of supernatant is transferred from the sample block in position C1 to the lysis plate in position B1. 440 µl of DX Binding with DX Binding Additive is added to the lysis plate. The lysate is then mixed 8 × and incubated at room temperature for 5 min. 600 µl of the lysate is added into the capture plate (Pre-mixed 8 ×). A vacuum of 35 kPa is applied for 5 min. 200 µl of DX Binding with DX Binding Additive is loaded into the capture plate. A vacuum of 35 kPa is applied for 5 min. 600 µl of DX Wash is loaded into the capture plate. A vacuum of 25 kPa applied for 1 min, repeated (2 iterations). 600 µl of DX Final Wash is loaded into the capture plate. A vacuum of 35 kPa is applied for 1 minute. A vacuum of 25 kPa is applied for 5 min to dry the plate. The carriage is moved to elution chamber. 200 µl of Elution buffer is loaded into the capture plate. The sample is then incubated for 5 min. A vacuum of 35 kPa is applied for 1 min.

We employed the QIAxtractor Software application. The tube was frozen at -20 °C for long-time preservation.

Fragments of four different mitochondrial genes were amplified by polymerase chain reaction (PCR): 12S rDNA, COI, cyt *b* and D-Loop (Table 1). We employed primers commonly used for fish published by Palumbi (1996), Ward et al. (2005), Kocher et al. (1989) and Lee et al. (1995) respectively. Amplification reactions were performed in a total volume of 23 µl, including 5 PRIME Buffer 1 × (Gaithersburg, MD, USA), 1.5 mM MgCl_2_, 0.25 mM dNTPs, 1 µM of each primer, 20 ng of template DNA, and 1.5U of DNA Taq polymerase (5 PRIME).

**Table 1.** Species considered within each case study; common and specific names and classification. Numbers of nucleotides obtained for each mtDNA gene fragment (length in bp) and GenBank Accession Numbers.

REGION	SPECIES		CLASSIFICATION (Order, Family)	Mitochondrial regions (length in bp)	GenBank A.N.
	Common name	Scientific name			
Amazon River	curimata	*Prochilodus nigricans*	Characiformes, Curimatidae	12S rDNA (380)	JN007487–JN007496
				COI (605)	JN007727–JN007734; HM480806–HM480807
				cyt *b* (293)	JN007647–JN007656
				D–Loop (424)	JN007567–JN007576
	jaraquí	*Semaprochilodus insignis*	Characiformes, Curimatidae	12S rDNA (380)	JN007497–JN007506
				COI (605)	JN007735–JN007744
				cyt *b* (293)	JN007657–JN007666
				D–Loop (424)	JN007577–JN007586
Cantabric Sea	mackerel	*Scomber scombrus*	Perciformes, Scombridae		
				12S rDNA (382)	JN007507–JN007516
				COI (605)	JN007745–JN007751; HM480797; HM480799; HM480819
				cyt *b* (293)	JN007667–JN007676
				D–Loop (412)	JN007587–JN007596
	tuna	*Thunnus alalunga*	Perciformes, Scombridae	12S rDNA (382)	JN007517–JN007526
				COI (605)	JN007752–JN007761
				cyt *b* (293)	JN007677–JN007687
				D–Loop (412)	JN007597–JN007606
Mediterranean Sea	anchovy	*Engraulis encrasicolus*	Clupeiformes, Engraulidae	12S rDNA (384)	JN007527–JN007536
				COI (605)	JN007762–JN007768; HM480814–HM480816
				cyt *b* (293)	JN007687–JN007696
				D–Loop (462)	JN007607–JN007616
	mackerel	*Scomber scombrus*	Perciformes, Scombridae	12S rDNA (384)	JN007537–JN007546
				COI (605)	JN007769–JN007777; HM480797
				cyt *b* (293)	JN007697–JN007706
				D–Loop (462)	JN007617–JN007626
Narcea River	Atlantic salmon	*Salmo salar*	Salmoniformes, Salmonidae	12S rDNA (439)	JN007547–JN007556
				COI (635)	JN007778–JN007787
				cyt *b* (322)	JN007707–JN007716
				D–Loop (460)	JN007627–JN007636
	brown trout	*Salmo trutta*	Salmoniformes, Salmonidae	12S rDNA (439)	JN007557–JN007566
				COI (635pb)	JN007788–JN007797
				cyt *b* (322)	JN007717–JN007726
				D–Loop (460)	JN007637–JN007646

The PCR conditions were the following:

12S rDNA: an initial denaturation at 95 °C for 10 min, then 35 cycles of denaturation at 94 °C for 1 min, annealing at 57 °C for 1 min and extension at 72 °C for 1.5 min, followed by a final extension at 72 °C for 7 min.

COI: an initial denaturation at 94 °C for 5 min, then 10 cycles of denaturation at 94 °C for 1 min, annealing at 64–54 °C for 1 min and extension at 72 °C for 1.5 min, followed by 25 cycles of denaturation at 94 °C for 1 min, annealing at 54 °C for 1 min and extension at 72 °C for 1.5 min, finally a final extension at 72 °C for 5 min.

cyt *b*: an initial denaturation at 94 °C for 5 min, then 10 cycles of denaturation at 94 °C for 1 min, annealing at 60–50 °C for 1 min and extension at 72 °C for 1.5 min, followed by 25 cycles of denaturation at 94 °C for 1 min, annealing at 54 °C for 1 min and extension at 72 °C for 1.5 min, finally a final extension at 72 °C for 5 min.

D-Loop: an initial denaturation at 94 °C for 5 min, then 10 cycles of denaturation at 94 °C for 1 min, annealing at 57 °C for 1 min and extension at 72 °C for 1.5 min, followed by 25 cycles of denaturation at 94 °C for 1 min, annealing at 54 °C for 1 min and extension at 72 °C for 1.5 min, finally a final extension at 72 °C for 5 min.

Sequencing was carried out by the DNA sequencing service GATC Biotech (Germany).

### Sequence edition

Sequences were visualized and edited employing the BioEdit Sequence Alignment Editor software (Hall 1999). Sequences were aligned with the MEGA v4.0 software (Tamura et al. 2007).

Putative proteins (amino acid sequences) from the COI and cyt *b* sequences were inferred with the software MEGA v4.0 (Tamura et al. 2007).

### Species identification from DNA sequences

The sequences obtained were compared with those existing in the public database GenBank using the BLAST tool (http://blast.ncbi.nlm.nih.gov/Blast.cgi?PROGRAM=blastn&BLAST_PROGRAMS=megaBlast&PAGE_TYPE=BlastSearch). Species were  identified based on maximum BLAST scores with matching sequences, corresponding to 100% coverage and 100% identity. When the haplotype was new (i.e. not present in GenBank and BOLD), a 100% coverage with 99% identity, or in a few cases 98% identity, was found for the matching sequence. COI barcodes were also compared against the BOLD database, uploading them in the BOLD identification system in FASTA format at http://www.boldsystems.org/index.php/IDS_OpenIdEngine. The system retrieves matching sequences with the corresponding % similarity (matching nucleotides) and gives the most likely species for the query sequence. If matching sequences from more than one species are retrieved with a similar probability, then the system displays all the possible putative species the query can be assigned to.

The two databases were accessed for species identification in September 2013.

### Diversity indices

Three well-known diversity indices were employed: number of haplotypes, haplotype diversity and nucleotide diversity. They were calculated with the DnaSP software (Librado and Rozas 2009). The same program was employed to generate concatenated data files with the different markers analyzed and re-estimate genetic diversity parameters.

Haplotype diversity is a measure of population variation, as the probability of two randomly chosen haplotypes in the sample being different. It is calculated with the formula described by Nei and Tajima (1981).

Nucleotide diversity indicates how different sequences are to each other. Its value is higher when sequences belong to distant taxa. It is defined as the average number of nucleotide differences per site between any two DNA sequences chosen randomly from the sample population, and is symbolised as π (Nei and Li 1979).

We have also used the simplest diversity measure Nh/n (number of haplotypes divided by the number of samples analysed).

### Statistical analysis

Comparison between genes for their polymorphic content was made based on means and variances of diversity parameters. It was performed using the software SPSS 13.0 software (SPSS Inc., Chicago, IL, USA).

## Results

### Species identification of the considered samples

For three study areas, the two most harvested species belonged to the same family (Table 1), viz. Curimatidae, Salmonidae and Scombridae in the Amazon River, Narcea River and Cantabric Sea, respectively. In the Mediterranean Sea, the two most harvested species were respectively anchovy *Engraulis encrasicolus* (Engraulidae) and mackerel *Scomber scombrus* (Scombridae).

PCR yielded positive amplifications in all cases, and sequences of different length were obtained for each marker and species analyzed: 380–439, 605–635, 293–322, 412–462 base pairs (bp) for 12S rDNA, COI, cyt *b* and D-Loop respectively (Table 1). The concatenated sequences were thus 1692–1856 bp long. The sequences obtained were submitted to the GenBank where they are available with the accession numbers reported in Table 1.

Clear and unambiguous species identification from significant matches with the databases was not always possible (Table 2). All the 12S rDNA sequences yielded a 100% identity score with at least one GenBank reference sequence (other than those generated in the present study) belonging to only one species, and were hence considered as being unambiguously identified. However, the results were less clear for the other genes and also varied among species. All mackerel samples were well-identified by the four genes and the two databases, whereas tuna retrieved more than one species with identical scores or match probabilities (*Thunnus alalunga*, *Thunnus thynnus* and *Thunnus orientalis*) for all cyt *b* and many COI and D-Loop sequences (Table 3). One D-Loop sequence retrieved *Thunnus albacares* as the closest match (Table 3). Ambiguous results (more than one putative species) were obtained from BOLD also for anchovy (COI sequences assigned to any of *Engraulis encrasicolus*, *Engraulis eurystole*, *Engraulis australis* and *Engraulis japonicus* species), brown trout (assigned indistinctly to *Salmo trutta* and *Salmo ohridanus* by BOLD), curimatá (*Prochilodus nigricans*, *Prochilodus rubrotaeniatus*, *Prochilodus lineatus*, *Prochilodus costatus*) and jaraquí (*Semiprochilodus insignis*, *Semiprochilodus taeniurus*, *Curimata inornata*). In GenBank ambiguous COI species identifications occurred for five tuna haplotypes that yielded identical and maximum matching scores with *Thunnus alalunga* and *Thunnus orientalis* sequences, and for jaraquí (*Semaprochilodus insignis* and *Semaprochilodus taeniurus* sequences yielded identical and maximum matching scores with our haplotypes). For cyt *b* of jaraquí (Table 3) the problem was not ambiguity but lack of external reference sequences in GenBank, viz. all the sequences yielding > 91% matching scores with ours were from the present study, and the closest identity with an external sequence (91%, unlikely the same species for a conserved coding gene) occurred with the sequence AY791437 of *Prochilodus nigricans*.

**Table 2.** Species identification based on the assayed genes in the four considered catches, measured as the number of individuals that are unambiguously assigned to a species in GenBank (all genes) and BOLD (COI). Databases accessed in September 2013.

	COI		12S rDNA	cyt *b*	D-Loop
	GenBank	BOLD	GenBank	GenBank	GenBank
**Cantabric Sea**					
mackerel	10	10	10	10	10
tuna	5	0	10	0	6
% catch	75%	50%	100%	50%	80%
**Mediterranean Sea**					
anchovy	10	0	10	10	10
mackerel	10	10	10	10	10
% catch	100%	50%	100%	100%	100%
**Narcea River**					
Atlantic salmon	10	10	10	10	10
brown trout	10	0	10	10	10
% catch	100%	50%	100%	100%	100%
**Amazon River**					
curimatá	10	0	10	10	10
jaraquí	0	0	10	0	10
% catch	50%	0%	100%	50%	100%

**Table 3.** Ambiguous or inconclusive matches between sequences in this study and reference sequences in GenBank (all sequences) and BOLD (COI). The species retrieved from each database (with maximum score for GenBank) are presented. + : Sequences for which there are > 5 entries in GenBank with a maximum score.

	GenBank	BOLD
Sequences of this study	COI	
JN007753,54,59,60,61	*Thunnus alalunga*	*Thunnus alalunga*, *Thunnus orientalis*, *Thunnus obesus*, *Thunnus thynnus*, *Thunnus atlanticus*
JN007752,55,56,57,58	*Thunnus alalunga*, *Thunnus thynnus*	*Thunnus alalunga*, *Thunnus orientalis*, *Thunnus obesus*, *Thunnus thynnus*, *Thunnus atlanticus*
HM480814–15, JN007765–68	*Engraulis encrasicolus*	*Engraulis encrasicolus*, *Engraulis eurystole*, *Engraulis australis*
HM480816, JN007762–64	*Engraulis encrasicolus*	*Engraulis encrasicolus*, *Engraulis capensis*, *Atherina breviceps*
JN007788 +	*Salmo trutta*	*Salmo trutta*, *Salmo ohridanus*
JN007727 +	*Prochilodus nigricans*	*Prochilodus nigricans*, *Prochilodus rubrotaeniatus*
JN007743 +	*Semaprochilodus insignis*, *Semaprochilodus taeniurus*	*Semaprochilodus insignis*, *Semaprochilodus taeniurus*, *Curimata inornata*
	**cyt *b***	
JN007677 +	*Thunnus alalunga*, *Thunnus orientalis*	
JN007657 +	None out of this study	
	**D-Loop**	
JN007604	*Thunnus albacares*	
JN007600–02	*Thunnus alalunga*, *Thunnus thynnus*	

### Genetic diversity in the four analyzed case studies

As expected, the four DNA regions exhibited different degrees of variability (Table 4). The non-coding D-Loop (58 haplotypes in total) was more variable than the two protein coding loci (31 and 27 haplotypes for cyt *b* and COI respectively) and the ribosomal 12S rDNA gene (15 haplotypes). The four marine species, the Amazonian jaraquí (*Semiprochilodus insignis*) and the north Spanish brown trout (*Salmo trutta*) exhibited ten different haplotypes in total considering the concatenated mitochondrial sequences analyzed. Fewer haplotypes were obtained for the Amazonian *Prochilodus nigricans* (6 haplotypes) and the Spanish *Salmo salar* (two haplotypes). In this latter species polymorphism occurred in the 12S rDNA gene, but not in the D-Loop, which was the most variable region in the other species. Overall nucleotide diversity was higher for marine than for freshwater settings for all markers as well as the concatenated sequence (Table 4). The highest Hd for both 12S rDNA and COI genes corresponded to the Amazonian samples, whereas marine catches were most variable at the less conserved cyt *b* and especially at the D-Loop. The least diverse Narcea River exhibited higher Hd at the highly conserved 12S rDNA than the two marine catches, due to Atlantic salmon polymorphism (likely due to a mixture of lineages remaining from past stocks transfers from North European populations; e.g. Horreo et al. 2011b).

**Table 4.** Sequence diversity in each species. Nh, Hd and π are the number of haplotypes, haplotype diversity and nucleotide diversity, respectively.

Locus	Parameter	Species							
		anchovy	mackerel (Cant.)	mackerel (Med.)	curimatá	*A. salmon*	brown trout	jaraquí	tuna
12S rDNA	Nh	2	1	2	2	2	2	3	1
n = 380-439	Hd	0.2	0	0.467	0.467	0.356	0.356	0.378	0
	π	0.052	0	0.124	0.123	0.081	0.081	0.105	0
COI	Nh	2	4	5	4	1	2	3	6
n = 605-635	Hd	0.2	0.533	0.8	0.733	0	0.556	0.689	0.778
	π	0.165	0.265	1.249	0.154	0	0.088	0.136	0.191
cyt *b*	Nh	3	4	8	1	1	5	6	3
n = 293-322	Hd	0.378	0.533	0.956	0	0	0.822	0.778	0.689
	π	0.205	0.273	1.82	0	0	0.469	0.394	0.88
D-Loop	Nh	8	10	10	6	1	5	8	10
n = 412-462	Hd	0.978	1	1	0.867	0	0.867	0.956	1
	π	1.893	2.126	3.655	0.65	0	0.358	1.268	6.362
All coding	Nh	4	6	10	5	2	8	8	7
n = 1278-1396	Hd	0.533	0.778	1	0.8	0.356	0.956	0.956	0.911
	π	0.141	0.188	1.048	0.111	0.025	0.174	0.186	0.293
All loci	Nh	10	10	10	6	2	10	10	10
n = 1682-1856	Hd	1	1	1	0.867	0.356	1	1	1
	π	0.588	0.644	1.738	0.244	0.019	0.219	0.449	1.744

**Figure 1.**
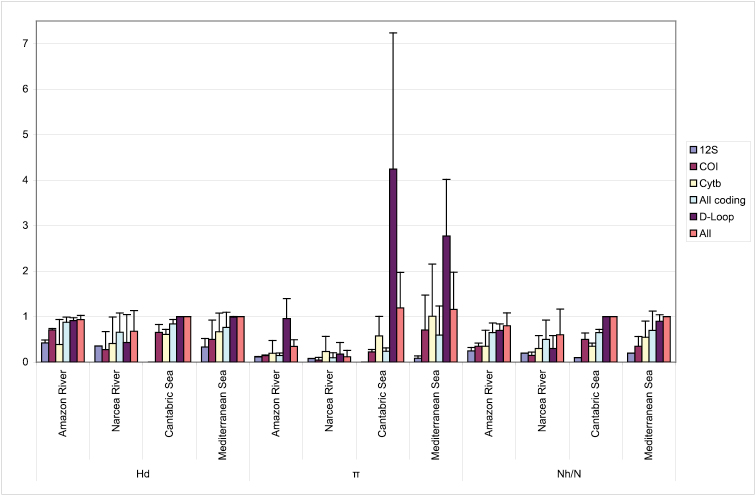
Summary of population genetic diversity retrieved fromeach mitochondrial region separately (12S rDNA, COI, cyt *b*, D-Loop), from the coding and from all regions concatenated (All), in the four case studies. Mean (standard deviation as vertical bars) is provided for Nh/n, Hd and π (mean number of different haplotypes per species, haplotype diversity and nucleotide diversity respectively).

The trade-off between using the same genetic analysis for simultaneously authenticating specimens and rapidly evaluating population diversity is that conserved species-specific sequences may not exhibit enough polymorphism. This is exemplified in Figure 1 and in the total number of variants of each marker found in this study, with 58 D-Loop versus only 15 12S rDNA haplotypes. Comparison between DNA regions for polymorphic information – measured as mean variation for each gene as in Figure 1 – yielded, despite small sample sizes, highly significant differences for all parameters when the six sequences were considered at the same time (p = 0.011, p = 0.006 and p = 0.000, for Hd, π and Nh/n, respectively). Most polymorphisms were provided by the non-coding D-Loop (Figure 1), and adding more nucleotides (concatenated sequence of all loci) did not increase significantly the level of polymorphism (p = 0.639, p = 0.109 and p = 0.428, for Hd, π and Nh/n, respectively). As expected, in relation with its length, the D-Loop was the most informative gene for quantifying diversity.

## Discussion

The results presented in this study illustrate how genetic methodologies could be applied in practice for monitoring fish catches. They also suggest some caveats of the current databases that should be considered in order to improve their built-in tools for species identification, especially if massive sequencing is envisaged. We have found ambiguous catch identifications in several cases. This is due to the fact that some identical haplotypes (sequences) are labeled in the databases with different specific names. Duplicated names at species level are a problem well recognized in reference databases such as GenBank (e.g. Federhen 2012). In this sense, we encourage a thorough taxonomic revision of the existing databases. The joint work of taxonomists and molecular systematists will help in the effort of cataloguing collections and voucher specimens (Puillandre et al. 2012). It may also happen that very closely related species share haplotypes at highly conserved genes. This could be the case of the *Thunnus* species, which are so closely related that they even give inconsistent phylogenetic signals (e.g. Chow and Kishino 1995). Mitochondrial introgression between species has been reported for this genus (Chow et al. 2006), so mitochondrial markers would not be a good choice for identifying tuna species. However, there was no ambiguity with the highly conserved 12S rDNA. Therefore, using this region may solve the problem in *Thunnus*. Although DNA barcoding through COI resolves most species, some taxa have proved intractable (Waugh 2007). We cannot explain what the reason was for all the cases found here, but it is clear that ambiguous identification would be a problem in routine large-scale fisheries barcoding. As also suggested by other authors (e.g. Savolainen et al. 2005, Austerlitz et al. 2009), incorporating nuclear genes as barcodes could help to solve these problems.

On the other hand, analyzing two DNA regions of different level of variability and recording simple polymorphism data in a database are easy actions that can be done very fast employing massive sequencing methodologies. They would hopefully allow to ascertaining the species and early detecting variation losses in catch. In a moment of stock overexploitation (Myers and Worm 2005) and urgent need of a better fisheries control in many regions (Worm and Branch 2012), these two issues are of most importance for long-term fisheries sustainability (Dahl 2000, Wessells et al. 2001, Pauly et al. 2002). For mitochondrial (haploid) sequences, simple statistical parameters for measuring sequence variation such as haplotype and nucleotide diversity could be incorporated into next-generation sequencing software, making it easier the process of diversity monitoring in fish landings. Hence, we propose to incorporate DNA barcoding as a first-instance routine surveys and periodical monitoring of catch diversity, but adding nuclear genes seems to be necessary (Markmann and Tautz 2005, Monaghan et al. 2005, Savolainen et al. 2005). If a decrease of variation is detected, further studies should follow, may be employing population genomics approaches and other biological tools. Diversity can be properly measured by using a diversity of tools and characters (Rubinoff 2006). Morphology (Wiens 2004), ecology (Crandall et al. 2000), adaptive differences (*sensu* Waples 1991) and genetic data from the mitochondrial and nuclear genomes, which can result in very different assessments of biodiversity, should be combined for having a complete perspective of the diversity of a community or ecosystem (Mouillot et al. 2011).

## Conclusions

Taking into account the number of existing sequences in databases, that is essential for species identification, and the polymorphic information provided by the different mitochondrial regions examined, the use of more than one gene and preferably a combination of nuclear and mitochondrial sequences would be recommended for routine genetic monitoring of fish catches. Incorporating new sequencing technologies will speed up large-scale genetic analysis of catch.
